# Complementary and alternative medicine use among infertile women attending infertility specialty clinics in South Korea: does perceived severity matter?

**DOI:** 10.1186/s12906-019-2727-x

**Published:** 2019-11-06

**Authors:** Jung Hye Hwang, Yi Young Kim, Hyea Bin Im, Dongwoon Han

**Affiliations:** 1 Department of Obstetrics and Gynecology, Hanyang University College of Medicine, Seoul, South Korea; 2 Department of Global Health and Development, Graduate school, Hanyang University, Seoul, South Korea; 3 Institute of Health Services Management, Hanyang University, Seoul, South Korea; 4 Graduate School of Public Policy, Hanyang University, Seoul, South Korea; 5 Department of Preventive Medicine, Hanyang University College of Medicine, 222 Wangsimni-ro, Seongdong-gu, Seoul 04763 South Korea

**Keywords:** Infertility, Reproductive health, Complementary and alternative medicine, Perceived severity of illness, Korea

## Abstract

**Background:**

Complementary and alternative medicine (CAM) use among infertile women is popular in many countries, including Korea. Previous research has repeatedly found more than half of infertile women surveyed use CAM therapies for fertility enhancement and overall well-being. However, there is currently little evidence to support this practice, and this raises the question of infertile women’s experiences in the uptake of those modalities and sociodemographic and psychological factors associated with CAM use. Thus, this study aims to explore the perceptions and experiences of infertile women with regard to their use of CAM in Korea.

**Methods:**

A cross-sectional study was conducted using data from 263 infertile women attending infertility specialty clinics in Seoul, Korea, in June 2012. The survey instrument included 47 questions on the use of CAM, demographic characteristics, health status, and infertility related factors such as duration and type of infertility, experience and satisfaction of conventional treatment, and self-perceived severity of infertility condition. Chi-square test and logistic regression were used for data analysis.

**Results:**

Among 286 respondents (response rate, 95.3%), a total of 263 women were included in the final analysis. 63.5% of respondents used one or more types of CAM modalities during infertility treatment. The utilization of CAM was associated with employment status, duration of infertility treatment, and self-perceived severity of the disease. The most commonly used CAM modalities were multivitamin and herbal medicine, and differences in types of CAM modalities used were found between the group with a higher rating of self-perceived disease severity and the lower perceived severity group.

**Conclusions:**

High prevalence of CAM use among infertile women was observed in Korea. Our findings support that infertile women’s own understanding of their illness and physical condition influences self-care behavior such as CAM use. This calls for an urgent need for further in-depth study of the clinical effects of popular CAM modalities among infertile women when used in conjunction with conventional treatment.

## Background

Complementary and alternative medicine (CAM) refers to a group of non-mainstream healthcare systems, practices, and products used either in conjunction with or in place of conventional medicine [1]. According to National Center for Complementary and Integrative Health (NCCIH), CAM modalities are categorized into three major groups: natural products, mind and body practices, and other complementary health approaches [1], but detailed categorization can vary depending on sociocultural background [2, 3]. A high prevalence of CAM use is especially seen in the reproductive age group [4, 5], where their main purpose of CAM use is for pregnancy-related health issues due to the perceptions that CAM modalities are safer, more natural, and holistic approach to health [6–9].

Among reproductive health concerns, infertility in particular, is a multifactorial chronic condition which affects 48.5 million couples worldwide [10]. The condition negatively affects physical, mental, and even social aspects of women in both developing and developed countries due to various factors such as late marriage, stress, obesity, long-term use of birth control, and pollution, yet it has not been receiving much attention [4, 11–17]. Korean women are also faced with challenges due to fertility-related issues: based on available data, 13.5% of women between the ages of 15 to 39 suffer from infertility [18], and 94.6% of those women suffer from depression. 26.6% of working women quit their jobs, and 8.9% take career-breaks to continue to receive infertility treatment [14]. For such reasons, infertility poses a great public health concern requiring an appropriate response plan.

It has been speculated that infertile women use CAM frequently in addition to conventional therapy not only to increase the chances of conception, but also to alleviate psychological, clinical, and physical concerns arising from being infertile and under various clinical treatments [4, 6, 7, 12, 19]. Although there is little evidence to support this practice, current international research suggests that 29–96% of infertile women seek CAM in addition to conventional therapies [11–13, 19–26], and this raises the question of infertile women’s experiences in uptake of these modalities, as well as sociodemographic and psychological factors associated with CAM use.

Nevertheless, even with such widespread use of CAM modalities, only a few studies have examined the pattern of CAM use among infertile women in Asian countries [27]. Furthermore, existing literature mainly focuses on examining the characteristics of CAM users, preferred modalities, source of information, and disclosure to the physician(s) [4]. A few studies have identified certain sociodemographic characteristics and health and illness-related characteristics to be correlated with CAM use [11, 12, 19, 28].

Among the factors associated with CAM uptake, we specifically focused on the self-perceived severity of the disease, a component of the Health Belief Model constructed by Rosenstock et al. [29]. Prior studies on chronic disease patients found a greater perception of disease severity to be associated with CAM use [30, 31], and other studies on women with reproductive concerns also found perceived symptom severity to be potential predictors of CAM use [32, 33]. Infertility also being a chronic condition [34] that requires relatively longer treatment duration, close monitoring, and continuous follow up of disease condition, we hypothesized that infertile women’s self-perceived severity of their condition to be associated with CAM use. Also, we speculated that differences would be found in the pattern of CAM use and clinical characteristics of infertile women with a greater perception of disease severity.

Hence, the aim of this study was not only to investigate general characteristics, but also to examine factors associated with CAM use among infertile women attending specialized infertility clinics in South Korea. Lastly, we examined differences in clinical, subjective well-being, and characteristics related to CAM use between women with a higher rating of self-perceived disease severity and those with a lower rating of disease severity.

## Methods

### Study setting and participants

This research was a descriptive cross-sectional in design, conducted at three specialized infertility clinics located in Seoul, South Korea. All infertile women visiting the clinics were eligible to take part in the survey. Exclusion criteria were women who were pregnant at the time of the survey and unable to speak or mentally unstable.

The sample size for this study was calculated using the formula for sample size determination using confidence interval (CI) of proportion: $n={z}_{\alpha /2}^2\bullet \frac{pq}{d^2}$. To determine the required sample size (n), the margin of error (d) was set on 0.06, and the estimated proportion of CAM use among infertile women (p) was calculated using the data from previous studies conducted in Korea (0.669) [35–37]. q is the proportion of people not using CAM (1-p), and *z*_0.025_ is 1.96, which corresponds to a confidence interval of 95%. This equation gave the minimum sample size as 236 participants, and we distributed a total of 300 survey questionnaires assuming 20% non-response rate.

### Survey instrument

After reviewing the literature [6, 11, 21], the survey questionnaire was developed in the Korean language. In order to examine content and face validation of the survey instrument, the questionnaire was reviewed by three researchers to assess the relevancy, organization, appropriateness, rationality, and clarity of the questions. A pilot study was also conducted on a sample of 20 participants to evaluate readability and ambiguities, and a few items were revised based on the results. The final version of the questionnaire consisted of 47 items, with both multiple-choice and open-ended questions, and respondents’ perceptions and attitudes were measured using a 5-point Likert scale.

The questionnaire consisted of four major sections. The first section had 14 questions concerning the respondents’ health status, the practice of health-promoting behavior [(practicing healthy diet, regular exercise, and adequate sleep (1 = never, 5 = very well)], presence of comorbidities, infertility related characteristics, gravidity, self-perceived severity of infertility condition (1 = not at all, 5 = very much), and the level of psychological stress (pressure and anxiety) due to infertility (1 = not at all, 5 = very much). The second section contained eight questions regarding patterns of health service use, satisfaction with infertility treatment, the experience of adverse reaction to conventional infertility treatment, and infertility treatment-related cost.

The third section included 18 questions on CAM use. The first question asked, “Did you use any CAM during the last 12 months?,” and the CAM users were asked to check which modalities were used from the list provided in the survey. The respondents who reported using at least one CAM modality were also asked about patterns of CAM use, reasons for use, perceived health benefits of CAM use, satisfaction with CAM, respondent’s attitudes and beliefs toward CAM, and source of information on CAM. The last section comprised of seven questions on participants’ age, level of income, level of education, occupation, and area of residence.

### Data collection

For this study, ethical approval of the study was obtained from the Institutional Review Board on Human Subjects Research and Ethics Committees, Hanyang University (HYI-12-019). Three surveyors were trained and recruited for data collection. Information about the research was given verbally to each respondent, and prior to the survey, all participants completed IRB-approved written informed consent. Participants were informed that they could quit the survey anytime they wanted to, and the respondents were asked to fill out the questionnaire themselves. A total of 300 survey questionnaires were distributed, but 14 participants did not return or complete the questionnaire (response rate: 95.3%). The data of 23 women who were pregnant at the time were also excluded from data analysis; therefore, the data of 263 respondents were included in the final analysis. The survey was conducted for eight days (starting from June 7th, 2012).

### Statistical analysis

The collected data of 263 were entered in Microsoft Excel and were analyzed using the software Statistical Package for Social Sciences (SPSS) v. 21. Descriptive statistics were used to describe sociodemographic and reproductive health-related characteristics of study respondents. Pearson’s Chi-square test and Fisher’s exact test were executed to identify differences in sociodemographic (age, education level, household income, religious affiliation, and employment status) and reproductive health-related characteristics (type of infertility, treatment duration, infertility diagnosis, type of current treatment, level of self-practice of health-promoting behavior, self-perceived stress and severity, effectiveness of conventional treatment, presence of comorbidity, and experiences of adverse reaction to conventional treatment) between users and non-users of CAM.

Also, to examine variations in types of CAM modalities used according to the perceived severity of infertility, we divided the respondents into two groups: severe and non-severe group based on self-rating of the severity of the disease. The high perceived disease severity group includes women who rated their severity as 4 or 5 on a 5-point Likert scale; the low perceived severity group includes women who rated the severity from 1 to 3. For data analysis, the categorization of CAM types was done by the National Center for Complementary and Integrative Health (NCCIH) domain: natural products, mind and body practices, and other complementary health approaches. However, all practices prescribed or performed by traditional Korean medicine doctors were classified as “other complementary health approaches.” Chi-square test was then performed to investigate differences in types of CAM modalities used based on self-rating of the severity of the disease.

Lastly, to examine potential factors associated with CAM use, significant factors from previous chi-square test (employment status, treatment duration, degree of the practice of health-promoting behavior, self-perceived severity of illness, the experience of adverse reaction to conventional treatment) were selected to be analyzed using a multivariate regression model. A *P*-value of less than 0.05 was considered statistically significant for all analyses.

## Results

### Sociodemographic characteristics of study participants

The sociodemographic characteristics of the study participants are presented in Table 1. The mean age of respondents was 34.4 years old (SD = 4.22). The majority were 30–39 years of age (76.8%), had higher than or equal to university education (88.6%), had a household income greater than 3 million KRW per month (57.1%), and were employed (54.4%). The proportion of employed women was significantly higher in the non-CAM user group (*p* = 0.012).

**Table 1:** Sociodemographic characteristics of participants

Characteristics	Overall *n* = 263 (%)	CAM users *n* = 167 (%)	Non-users *n* = 96 (%)	*P*-value
Mean age (years)	34.4 ± 4.22	34.5 ± 4.19	34.2 ± 4.30	
< 30	30 (11.4)	20 (12.0)	10 (10.4)	0.756^a^
30–34	112 (42.6)	67 (40.1)	45 (46.9)	
35–39	90 (34.2)	59 (35.3)	31 (32.3)	
≥ 40	31 (11.7)	21 (12.6)	11 (10.4)	
Education level				
≤ Secondary	30 (11.4)	19 (11.4)	11 (11.5)	0.984^a^
≥ University level	233 (88.6)	148 (88.6)	85 (88.5)	
Household income				
< 300 million KRW	51 (19.4)	34 (20.4)	17 (17.7)	0.569^a^
300–500 million KRW	116 (44.1)	76 (45.5)	40 (41.7)	
≥ 500 million KRW	96 (36.5)	57 (34.1)	39 (40.6)	
Religious affiliation				
Yes	179 (68.1)	118 (70.7)	61 (63.5)	0.307^a^
No	84 (31.9)	49 (29.3)	35 (36.5)	
Employment status				
Yes	143 (54.4)	81 (48.5)	62 (64.6)	0.012^a^
No	120 (45.6)	86 (51.5)	34 (35.4)	

*CAM* Complementary and Alternative Medicine

^a^ Denotes Pearson’s Chi-square tests

### Infertility characteristics of study participants

Table 2 shows the fertility-related characteristics of the study participants. The majority of respondents suffered from primary infertility (53.2%). 65.4% of respondents had been under infertility treatment for less than 2 years, and the difference in treatment duration between CAM user and non-user groups was statistically significant (*p* = 0.010). Female infertility was the most common diagnosis (27.0%), and 54.4% of respondents were under the IVF treatment.

**Table 2:** Fertility-related characteristics of participants

Characteristics	Total *n* = 263 (%)	CAM users *n* = 167 (%)	Non-users *n* = 96 (%)	*P*-value
Type of infertility				
1° infertility	140 (53.2)	88 (52.7)	52 (54.2)	0.351^a^
2° infertility	123 (46.8)	79 (47.3)	44 (45.8)	
Infertility treatment duration				
< 2 years	172 (65.4)	101 (60.5)	71 (74.0)	0.010^a^
2–4 years	57 (21.7)	46 (27.5)	11 (11.5)	
> 4 years	34 (12.9)	20 (12.0)	14 (14.6)	
Infertility diagnosis				
Female factor only	71 (27.0)	47 (28.1)	24 (25.0)	0.759^b^
Male factor only	22 (8.4)	14 (8.4)	8 (8.3)	
Male and female factors	15 (5.7)	10 (6.0)	5 (5.2)	
Don’t know	50 (19.0)	28 (16.8)	22 (22.9)	
Unknown (not identified)	95 (36.1)	63 (37.7)	32 (33.3)	
Others	10 (3.8)	5 (3.0)	5 (5.2)	
Type of treatment				
Non-IVF treatment	120 (45.6)	73 (43.7)	47 (49.0)	0.411^a^
IVF treatment	143 (54.4)	94 (56.3)	49 (51.0)	

*CAM* Complementary and Alternative Medicine

^a^ Denotes Pearson’s Chi-square tests

^b^ Denotes Fisher’s exact test

### Health-related characteristics of respondents

Health-related characteristics of the study participants are demonstrated in Table 3. The average score of health-promoting behavior (practicing a healthy diet, regular exercise, and adequate sleep) in the CAM user group was significantly greater than that of non-users (*p* = 0.025). The majority of respondents (81.4%) did not suffer from other comorbidities, and the mean score of the self-perceived severity was significantly higher among CAM users (*p* = 0.009). The respondents rated their self-perceived effectiveness of conventional treatment and their satisfaction level as 3.54 (SD = 0.69) and 3.59 (SD = 0.63), respectively. 75.7% of respondents had no experience of adverse reaction to conventional infertility treatment; however, the proportion of women who experienced an adverse reaction to conventional treatment was greater in the CAM user group (*p* = 0.028).

**Table 3:** Health-related characteristics of respondents (*n* = 263)

Characteristics	Overall *n* = 263 (%)	CAM users *n* = 167 (%)	Non-users *n* = 96 (%)	*P*-value
Practice of health-promoting behavior (Mean ± SD)^#^	3.02 ± 0.68	3.10 ± 0.65	2.86 ± 0.72	
Yes (4,5)	52 (19.8)	40 (24.0)	12 (12.5)	0.025^a^
No (1,2,3)	211 (80.2)	127 (76.0)	84 (87.5)	
Presence of comorbidity				
Yes	49 (18.6)	32 (19.2)	17 (17.7)	0.870^a^
No	214 (81.4)	135 (80.8)	79 (82.3)	
Self-perceived stress (Mean ± SD)*	3.77 ± 0.83	3.85 ± 0.81	3.64 ± 0.86	
Severe (4,5)	158 (60.1)	107 (64.1)	51 (53.1)	0.081^a^
Not severe (1,2,3)	105 (39.9)	60 (35.9)	45 (46.9)	
Self-perceived severity of their condition (Mean ± SD)°	3.72 ± 0.90	3.78 ± 0.84	3.61 ± 0.98	
Not severe (1,2,3)	105 (39.9)	57 (34.1)	48 (50.0)	0.009^a^
Severe (4)	105 (39.9)	78 (46.7)	27 (28.1)	
Very severe (5)	53 (20.2)	32 (19.2)	21 (21.9)	
Self-perceived effectiveness of conventional treatment (Mean ± SD)^1^	3.54 ± 0.69	3.51 ± 0.65	3.60 ± 0.76	
Effective (4,5)	134 (51.0)	83 (49.7)	51 (53.1)	0.593^a^
Ineffective (1,2,3)	129 (49.0)	84 (50.3)	45 (46.9)	
Satisfaction level of conventional treatment (Mean ± SD)^2^	3.59 ± 0.63	3.56 ± 0.60	3.63 ± 0.68	
Satisfied (4,5)	147 (55.9)	91 (54.5)	56 (58.3)	0.546^a^
Dissatisfied (1,2,3)	116 (44.1)	76 (45.5)	40 (41.7)	
Experience of adverse reaction to conventional treatment				
Yes	64 (24.3)	48 (28.7)	16 (16.7)	0.028^a^
No	199 (75.7)	119 (71.3)	80 (83.3)	

^#^ Participants were asked to rate their practice of health-promoting behavior (practicing a healthy diet, regular exercise, and adequate sleep) (1 = Never, 5 = Always)

*Participants were asked to rate their self-perceived stress level (1 = Not at all, 5 = Very much)

°Participants were asked to rate their self-perceived severity of infertility condition (1 = Not at all, 5 = Very much)

^1^Participants were asked to rate their self-perceived effectiveness of conventional treatment (1 = Not at all, 5 = Very much)

^2^Respondents were asked to rate their satisfaction to conventional fertility treatment (1 = Very dissatisfied, 5 = Very satisfied)

^a^ Denotes Pearson’s Chi-square tests

### CAM modalities used based on the perception of disease severity

Table 4 presents the CAM modalities used by respondents based on the self-rating of disease severity. Among natural products, multivitamin was most used modality (84.4%) followed by Korean black raspberry (Rubus coreanus). Consumption of multivitamin (*p* = 0.043), Korean black raspberry (*p* = 0.008), ginseng and red ginseng (*p* <  0.001), blood circulation supplement pills (*p* = 0.006), and herbal tea (*p* = 0.013) was significantly greater in the group with a higher rating of disease severity. For mind and body practices, practice of lower body and hot spring bath, yoga, and pray/meditation was most common among respondents (31.7, 25.9, 15.6% respectively), and it was also found that practice of lower body and hot spring bath (*p* = 0.028) was significantly higher in the group with the greater perception of disease severity. Among traditional Korean medicine approaches, consumption of herbal medicine was most popular (72.5%), followed by moxibustion (19.2%), and a statistically significant difference was shown in the use of herbal medicine (*p* = 0.001).

**Table 4:** CAM modalities used by infertile women based on their perceived level of disease severity

CAM modalities	Overall *n* = 167 (%)	High perceived severity (4,5) ^#^ *n* = 110 (%)	Low perceived severity (1,2,3) ^#^ *n* = 57(%)	*P*-value
Natural Product*				
Multivitamin (folic acid, iron supplement, etc.)	141 (84.4)	93 (84.5)	48 (84.2)	0.043^a^
Korean black raspberry (Rubus coreanus/Rubi Fructus)	74 (44.3)	54 (49.1)	20 (35.1)	0.008^a^
Ginseng and red ginseng	63 (37.7)	50 (45.5)	13 (22.8)	< 0.001^a^
Mugwort	36 (21.6)	26 (23.6)	10 (17.5)	0.143^a^
Brown rice	46 (27.5)	30 (27.3)	16 (28.1)	0.509^a^
Blood circulation supplement pills	15 (9.0)	14 (12.7)	1 (1.8)	0.006^b^
Herbal tea	9 (5.4)	9 (8.2)	0 (0.0)	0.013^b^
Mind and body practices*				
Lower body bath/ hot spring bath	53 (31.7)	39 (35.5)	14 (24.6)	0.028^a^
Yoga	45 (25.9)	31 (28.2)	14 (24.6)	0.242^a^
Pray/meditation	26 (15.6)	18 (16.4)	8 (14.0)	0.400^a^
Psychotherapy	6 (3.6)	6 (5.5)	0 (0.0)	0.084^b^
Hypogastric breathing	4 (2.4)	1 (0.9)	3 (5.3)	0.305^b^
Chiropractic	3 (1.8)	3 (2.7)	0 (0.0)	0.278^b^
Massage	15 (9.0)	12 (10.9)	3 (5.3)	0.173^b^
Magnetic therapy	3 (1.8)	1 (0.9)	2 (3.5)	0.566^b^
Other complementary health approaches (Korean traditional medicine)*				
Herbal medicine	121 (72.5)	86 (78.2)	35 (61.4)	0.001^a^
Moxibustion	32 (19.2)	23 (20.9)	9 (15.8)	0.179^a^
Acupuncture	23 (13.8)	18 (16.4)	5 (8.8)	0.075^b^
Bee venom therapy	8 (4.8)	6 (5.5)	2 (3.5)	0.483^b^

* Columns do not add up to 100% due to use of multiple treatments

^#^ Perceived severity of disease was self-rated by participants on a 5-point Likert scale (1 = Not at all, 5 = Very much). Those who rated their severity from 1 to 3 were categorized as ‘low perceived severity’ group, whereas those who scored from 4 to 5 were put in ‘high perceived severity’ group

^a^ Denotes Pearson’s Chi-square tests

^b^ Denotes Fisher’s exact test

### Clinical characteristics of respondents by perceived severity of the disease

Clinical characteristics of respondents based on self-rating of the severity of the disease are shown in Table 5. The number of respondents with higher self-perceived stress was significantly greater in the high perceived severity group (*p* <  0.001). The proportion of women with infertility treatment duration greater than 2 years was also significantly higher in the group with a greater perception of disease severity (*p* <  0.001). Furthermore, significant differences in the time since diagnosis of infertility were observed (*p* = 0.001), where 56.9% of women in high-perceived severity group have been diagnosed with infertility longer than 2 years while 34.3% of women in low perceived severity group had the diagnosis for greater than 2 years.

**Table 5:** Clinical characteristics of respondents by perceived severity of disease (*n* = 263)

Characteristics	Overall *n* = 263 (%)	High perceived severity *n* = 158 (%)	Low perceived severity *n* = 105 (%)	*P*-value
Self-perceived stress (Mean ± SD)*	3.77 ± 0.83	4.16 ± 0.69	3.18 ± 0.68	
Severe (4,5)	158 (60.1)	132 (83.5)	26 (24.8)	< 0.001^a^
Not severe (1,2,3)	105 (39.9)	26 (16.5)	79 (75.2)	
Infertility treatment duration				
< 2 years	172 (65.4)	87 (55.1)	85 (81.0)	< 0.001^a^
2–4 years	57 (21.7)	44 (27.8)	13 (12.4)	
> 4 years	34 (12.9)	27 (17.1)	7 (6.7)	
Practice of health-promoting behavior (Mean ± SD)^#^	3.02 ± 0.68	3.04 ± 0.70	2.98 ± 0.65	
Yes (4,5)	52 (19.8)	34 (21.5)	18 (17.1)	0.431^a^
No (1,2,3)	211 (80.2)	124 (78.5)	87 (82.9)	
Time since diagnosis				
< 2 years	137 (52.1)	68 (43.0)	69 (65.7)	0.001^a^
2–4 years	69 (26.2)	50 (31.6)	19 (18.1)	
> 4 years	57 (21.7)	40 (25.3)	17 (16.2)	

*Participants were asked to rate their self-perceived stress level (1 = Not at all, 5 = Very much)

^**#**^ Participants were asked to rate their practice of health-promoting behavior (practicing a healthy diet, regular exercise, and adequate sleep) (1 = Never, 5 = Always)

^a^ Denotes Pearson’s Chi-square tests

### Potential predictors of CAM use

The result of multivariate logistic regression analysis is demonstrated in Table 6. From the multivariate model, non-employment (OR: 1.87, CI: 1.08–3.23), infertility treatment duration of 2–4 years (OR: 2.31, CI: 1.07–4.97), and self-perception of their disease severity as severe (OR: 2.14, CI: 1.16–3.95) were associated with CAM use.

**Table 6:** Multivariate logistic regression analysis between demographic, health behavior, and reproductive health-related factors and use of CAM

Characteristics	OR	95% CI	*P*-value
Employment status			
Yes	1	Ref	
No	1.87	1.08–3.23	0.025
Infertility treatment duration			
< 2 years	1	Ref	
2–4 years	2.31	1.07–4.97	0.033
> 4 years	0.90	0.40–2.01	0.793
Practice of health-promoting behaviorª			
No (1,2,3)	1	Ref	
Yes (4,5)	2.02	0.96–4.24	0.063
Self-perceived severity of their condition^b^			
Not severe (1,2,3)	1	Ref	
Severe (4)	2.14	1.16–3.95	0.014
Very severe (5)	0.93	0.45–1.96	0.856
Experience of adverse reaction to conventional treatment			
No	1	Ref	
Yes	0.64	0.33–1.25	0.193

ªParticipants were asked to rate their practice of health-promoting behavior (practicing a healthy diet, regular exercise, and adequate sleep) (1 = Never, 5 = Always)

^b^Participants were asked to rate their self-perceived severity of infertility condition (1 = Not at all, 5 = Very much)

## Discussion

This study explored the prevalence and determinants of CAM use among infertile women in South Korea. The findings of our study showed a high prevalence of CAM utilization by 63.5% of the participants. This usage was higher compared to the reports in Ireland (46%), Lebanon (41%), the U.S. (29%), and the U.K. (40%), yet lower than the prevalence found in Uganda (76%), Australia (66%), Taiwan (96%), and Turkey (82%) [11–13, 20–25]. These variations in prevalence can be explained by different definitions of CAM among studies, increase in CAM use over time, and actual differences in CAM use among respondents due to various cultural and socio-demographic characteristics [2].

Among sociodemographic and health-related characteristics of participants, a higher proportion of non-employed women were observed in the CAM user group, yet in contrast to earlier findings, no significant differences were found between CAM users and non-users in education level and household income [13, 19]. The proportion of infertile women with treatment duration longer than 2 years was significantly greater in the CAM user group, whereas the type of infertility and current treatment type did not differ between the two groups in our findings. Similar to a previous study, we found a higher proportion of women engaging in health-promoting behavior such as the practice of a healthy diet, sufficient exercise, and adequate sleep in the CAM user group [11]. More women who have experienced an adverse reaction to conventional infertility treatment were in the CAM user group, which can be attributed to mistrust in conventional medicine [38].

Congruent with previous findings in Turkey and Australia, the most commonly used CAM modality was multivitamin and other dietary supplements, while physical therapies such as chiropractic and qigong exercises were the least favored modalities [4, 23–25]. Patterns of CAM use reflect socioeconomic, cultural practices and beliefs [12, 22, 26], as indicated by the high prevalence of herbal medicine use (71.3%) among Korean women in our study due to the popular use of traditional Korean medicine [39]. Similarly, due to easy accessibility and availability of traditional medicine, the prevalence of herbal medicine use among Taiwanese infertile women was 96% [24], whereas the prevalence was lower in Middle Eastern countries such as Turkey (29.3%) and Lebanon (43.5%) [12, 25]. The popularity of herbal medicine use was also lower in western countries such as the U.S. (16%), Australia (29%), and Ireland (46%) [11, 20, 23]. However, in the Middle East, where religion is an essential part of their daily lives, a high prevalence of religious intervention was observed [12, 22, 25].

Among natural CAM products, other than a multivitamin, Korean black raspberry, also known as Rubi Fructus or Rubus coreanus, was found to be the most popular product among infertile women in Korea. In Donguibogam, a seventeenth-century Korean medical textbook upon which Traditional Korean Medicine has developed, Korean black raspberry is described as being effective in increasing male stamina and treating female infertility [40]. Based on this ancient tradition and cultural belief, it was observed that 44.3% of the respondents in our study had consumed Korean black raspberry to increase the chances of conception. This finding is particularly important as it demonstrates an example of popular use of non-evidence based medicine without proper physician consultation, and this calls for an immediate response plan as unsupervised use of traditional modalities may cause adverse effects when concurrently used with conventional medicine.

We further analyzed types of CAM modalities used depending on the level of self-rated disease severity, as it was found to be associated with CAM use in our study. Previous studies on women with reproductive concerns have found perceived symptom severity to be associated with CAM use [32, 33], yet no study has identified differences in the pattern of CAM use and clinical characteristics of infertile women with a greater perception of disease severity.

Under natural products, the differences between the two groups were statistically significant for the consumption of multivitamin, Korean black raspberry, ginseng and red ginseng, blood circulation supplement pills, and herbal tea. As for mind and body practices, the practice of lower body and hot spring bath was significantly higher in the high-perceived severity group. Among other complementary health approaches, the use of herbal medicine was significantly greater in the group with a higher rating of the perceived severity of the disease. Thus, significant differences in the pattern of CAM use found between the group with higher perceived severity and the lower perceived severity group were mostly in regards to using modalities with phyto-medicinal properties (herbal medicine, herbal tea, Korean black raspberry, and ginseng) rather than simple manipulative practices. Although believed to be safe, the evidence for the effectiveness of CAM use is still inconclusive, and some of the natural products and herbal medicine may have a negative effect on infertile women undergoing hormone-sensitive therapies [41–44]. Therefore, physicians should pay attention to patients’ use of CAM modalities during conventional treatment to prevent potential adverse effects when used in conjunction with conventional medical treatment, and additional studies should be conducted to ensure the safety and effectiveness of CAM modalities.

Differences in clinical and health-related characteristics of infertile women depending on the level of perceived disease severity were also observed in this study. Due to the financial and psychological burden which comes with treatment costs, social and cultural stigma, and emotional difficulties, notable consequences of infertility are anxiety, stress, and depression [45, 46]. Our findings also show a higher level of self-perceived stress in the group with a greater perception of disease severity. Furthermore, the proportion of women with infertility treatment duration longer than 2 years was greater in higher-rating of disease severity group, and the number of women who have been diagnosed with infertility for longer than 2 years was also higher in the group with greater disease severity. Thus, these emotional stressors may have led the women to seek additional care outside of conventional medical care despite the lack of definitive effects of CAM modalities [41, 47, 48].

Potential predictors of CAM use established in our sample include non-employment, infertility treatment duration of 2–4 years, and self-perception of disease severity as severe. Previous studies found household income, age of the respondent, type of infertility treatment, duration of the marriage, education level, positive CAM attitudes as possible predictors of CAM use, yet our results displayed no congruency [11, 12, 19, 28].

Limitations of this study include recall bias and lack of variability in research locations. The participants were selected from three infertility clinics located in Seoul city; therefore, the views and opinions of study participants may not fully reflect those of the other infertile women. However, despite these limitations, this study is the first study to examine differences in types of CAM modalities used and the clinical characteristics of respondents by the perceived severity of the disease.

## Conclusions

Our findings demonstrate that CAM use is common among infertile women in Korea. Those also highlight the association of CAM use with employment status and a reproductive health condition such as infertility treatment duration and self-perceived severity of infertility condition. Significant differences were also reported on the preferred method of CAM modalities between the group with a higher rating of self-perceived disease severity and lower perceived severity group. As such, infertile women’s own understanding and perception of their illness and physical condition influence self-care behavior such as CAM use. To provide the best care to infertile women, physicians must be recommended to stay up to date with knowledge on CAM use and the safety of CAM modalities, specifically when used in conjunction with conventional treatment.

## Data Availability

The data will be accessible by contacting the corresponding author of this study.

## Abbreviations

CAM: Complementary and alternative medicine
IUI: Intrauterine insemination
IVF: In vitro fertilization
